# Standardized Chromatographic and Computational Approaches for Lipophilicity Analysis of Five Gliflozin Antidiabetic Drugs in Relation to Their Biological Activity

**DOI:** 10.3390/molecules30010115

**Published:** 2024-12-31

**Authors:** Anna Gumieniczek, Anna Berecka-Rycerz, Marcelina Dul, Aleksandra Pryjda

**Affiliations:** Department of Medicinal Chemistry, Medical University of Lublin, Jaczewskiego 4, 20-090 Lublin, Poland; anna.berecka-rycerz@umlub.pl (A.B.-R.); marcelina.dul@gmail.com (M.D.); aleksandra.pryjda@onet.pl (A.P.)

**Keywords:** gliflozins, lipophilicity, chromatography, computational methods, chemometrics, pharmacological properties

## Abstract

This study represents the first-time experimental analysis of lipophilicity for antidiabetic drugs from the gliflozin class using chromatographic methods alongside computational approaches. The lipophilicity of five gliflozins (canagliflozin (CANA), dapagliflozin (DAPA), empagliflozin (EMPA), ertugliflozin (ERTU), and sotagliflozin (SOTA)) was assessed using R_MW_ and log k_W_ parameters with RP8, RP18, and CN coatings, while methanol and acetonitrile were used as organic modifiers. To enhance the reliability, six reference substances with established lipophilicity values (0.62–3.5) were used for standardization. For computational analyses, the methods ALOGP, iLOGP, MLOGP, SILICOS-IT, WLOGP, XLOGP3, and Consensus. Log P were applied. Descriptive statistics, correlation analyses, and chemometric techniques were employed to compare the results. Experimental lipophilicity values showed strong correlations, indicating that R_MW_ and log k_W_ are reliable parameters for evaluating the lipophilicity of these therapeutically valuable drugs. However, computational lipophilicity values were less consistent, both among themselves and compared to experimental data. Finally, the experimental lipophilicity of gliflozins was analyzed in relation to their pharmacological properties, including protein binding, renal clearance, volume of distribution, half-life, potency (IC_50_), and lipophilic ligand efficiency (LLE). Our results allow for a confident proposal of a model to experimentally determine the lipophilicity of gliflozin drugs including new derivatives.

## 1. Introduction

According to the IUPAC, lipophilicity refers to a molecule’s affinity for a lipophilic environment [1]. Lipophilicity is a key factor influencing drug absorption, distribution, transport, solubility in biological systems, stability, and acid–base properties. It also significantly affects drug interactions with receptors and other biological targets, as well as their potential toxic effects [2]. Traditionally, lipophilicity is measured using the shake-flask method, which determines the partition coefficient (log P) of a substance between organic and aqueous phases. However, this method has several drawbacks: it is time-consuming, requires large quantities of substances, and can lead to emulsion formation [3]. For these reasons, chromatographic methods, such as thin-layer chromatography (TLC) and high-performance liquid chromatography (HPLC), are widely used to assess lipophilicity. Other methods include chromatography with immobilized artificial membranes (IAMs), microemulsion electrokinetic chromatography (MEKC), and biopartitioning micellar chromatography (BMC) [3,4]. TLC and HPLC experiments frequently employ hydrophobic stationary phases in reversed-phase mode (RP18 and RP8), where retention is determined by hydrophobic interactions. Less hydrophobic adsorbents, such as CN, NH_2_, and DIOL, are also used, particularly for separating hydrophilic or charged substances, as they yield more reliable lipophilicity values than RP18 and RP8 phases [3,5]. In TLC, lipophilicity is estimated using the R_M_ parameter, calculated as follows:

R_M_ = log(1/R_F_ − 1) (1)

where R_F_ is the retention factor of the tested compound [6]. Boyce and Milborrow [7] introduced the R_MW_ parameter, i.e., an R_M_ value extrapolated to 100% water in the mobile phase according to the following equation:

R_M_ = R_MW_ − S × φ (2)

where φ represents the organic solvent’s volume fraction in the mobile phase, and S is the slope of the regression curve.

In HPLC, the chromatographic lipophilicity parameter is expressed as log k_W_, determined from the following equation:

log k = log k_W_ − S × φ (3)

where log k is the retention coefficient, calculated using retention time and dead retention time, and log k_W_ is the retention factor for a mobile phase containing 100% water, while φ and S represent the volume fraction of the organic solvent and the slope of the regression curve, respectively [3,4].

Chromatographic methods offer several advantages for lipophilicity assessment: they require minimal compound quantities, offer a wide range of stationary phases, and a robust understanding of solute interactions in chromatography [3,5]. TLC provides additional benefits, including the ability to test impure compounds and analyze multiple samples with varying lipophilicity in a single experiment. HPLC offers automation capabilities and is OECD-recommended [2,8,9]. Common mobile-phase mixtures for these methods include water with methanol, acetonitrile, or tetrahydrofuran, with dioxane and acetone used less frequently. Adding an organic modifier reduces the mobile phase’s polarity, increasing the solubility of compounds with high or moderate hydrophobicity [3,4,5].

Lipophilicity can also be calculated using various computational approaches, including molecular fragmentation, atomic, and whole-molecule property methods [10,11,12]. Multi-property prediction software is also used and integrates several computational models to predict log P alongside other ADME properties, often using hybrid models that combine empirical data, machine learning, and theoretical calculations [13]. However, these methods, being beneficial for rapid, high-throughput lipophilicity screening, each have limitations. For instance, fragment-based methods may struggle with novel or highly complex structures, while quantum chemical calculations are computationally intensive and generally limited to smaller molecules. Machine learning models require large, diverse training datasets and may not generalize well to compounds outside the training set [13].

**Figure 1:**
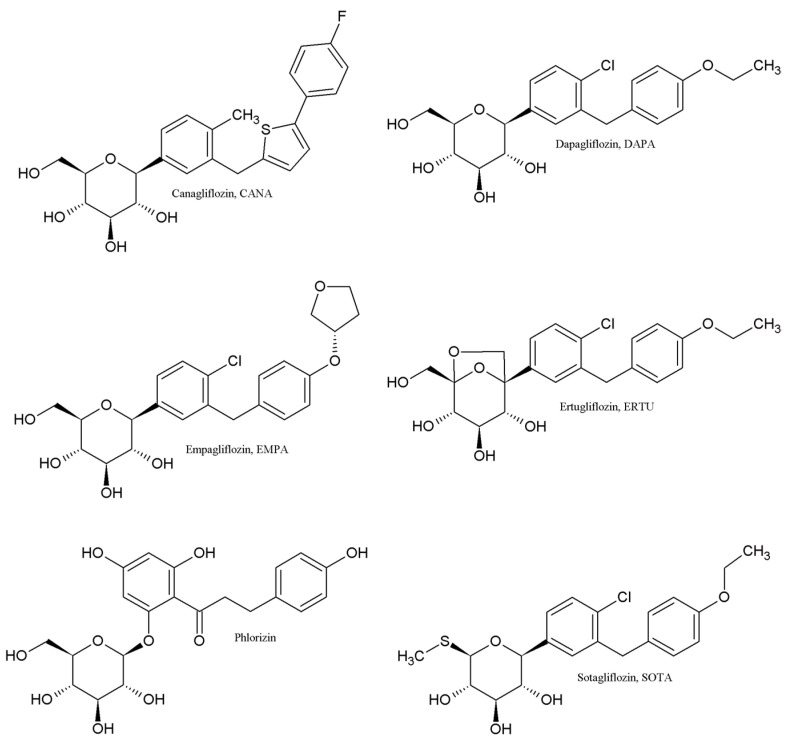
Chemical structures of the analyzed gliflozins: canagliflozin (CANA), dapagliflozin (DAPA), empagliflozin (EMPA), ertugliflozin (ERTU), and sotagliflozin (SOTA), and their lead structure phlorizin.

Gliflozins, inhibitors of sodium–glucose transporters (SGLTs), play a crucial role in glucose, amino acid, vitamin, and ion transport in the small intestine and renal tubules. Among SGLT proteins, SGLT1 is mainly involved in intestinal glucose absorption, while SGLT2 mediates glucose reabsorption in the kidney [14]. The initial design of gliflozins began with the phlorizin lead structure (Figure 1) and aimed to develop more stable O-glycosides. This effort ultimately yielded highly effective, stable C-analogs. The C-glycosidic ring provides SGLT inhibitory properties, while lipophilic group additions enhance SGLT2 selectivity. Structural features common to gliflozins include a methylene bridge linking various aryl and heteroaryl groups in their aglycones, with substituents such as an ethoxy group at position 4′ of the distal benzene and a chloro or methyl group at position 4 of the proximal benzene [15].

Despite gliflozins’ therapeutic relevance, experimental data on their individual lipophilicities are scarce, and only computational estimates of lipophilicity values have been reported to date. Limited research in this area may be due to the structural variations among gliflozins, complicating the selection of optimal experimental conditions. Hosny’s research examined lipophilicity in four antidiabetic drugs from different pharmacological classes, including two gliflozins, using normal-phase TLC. Although findings indicated that these two gliflozins are more lipophilic than metformin and linagliptin, no quantitative lipophilicity values were provided [16].

The present study aims to assess the lipophilicity of five gliflozin antidiabetic drugs, i.e., canagliflozin (CANA), dapagliflozin (DAPA), empagliflozin (EMPA), ertugliflozin (ERTU), and sotagliflozin (SOTA), using multiple TLC and HPLC chromatographic systems. This research explores optimal experimental conditions, including the impact of stationary phases, organic modifiers, and their concentrations. Additionally, we applied several computational algorithms, available on the SwissADME and VCCLAB platforms [17,18]. The obtained lipophilicity values were compared using correlation analyses, descriptive statistics, and chemometric techniques to identify similarities and differences. The most reliable lipophilicity values were then analyzed in relation to the key biological properties of these important drugs whose chemical structures are presented in Figure 1.

## 2. Results and Discussion

In the present study, a set of six well-characterized standards with known lipophilicity values ranging from 0.62 to 3.5 [19] and diverse structures were used. These compounds had been extensively investigated in our previous work for their applicability in chromatographic lipophilicity determination [20]. Our earlier experiments demonstrated that these standards exhibit measurable retention in different chromatographic systems and could be used for standardization. After chromatographic experiments and obtaining respective retention data (R_F_ and t_R_ values), the R_M_ and log k values for the tested gliflozin drugs, i.e., CANA, DAPA, EMPA, ERTU, and SOTA, were calculated alongside these six reference standards (Tables S1 and S2 in Supplementary Files). Respective values were then extrapolated to 100% water in the mobile phase, to obtain the R_MW_ and log k_W_ parameters using Equations (2) and (3). All resulting relationships demonstrated sufficient linearity, with determination coefficients (r^2^) exceeding 0.9 (Tables S3–S6 in Supplementary Files).

Interestingly, the R_MW_ and log k_W_ values for CANA, DAPA, EMPA, ERTU, and SOTA in the RP systems, specifically RP18 and RP8, were found to be higher when methanol was employed as the organic modifier compared to acetonitrile. However, this trend was not observed with the more polar CN phases, where no consistent differences emerged between these two solvents. Across all stationary phases, R_MW_ values obtained with the RP18 phase were generally higher than those observed with RP8 and CN coatings, with the exception of the values obtained for EMPA. In contrast, the log k_W_ values from the HPLC methods exhibited the least variability among RP18, RP8, and CN coatings (Tables S3–S6 in Supplementary Materials).

To further assess the lipophilicity of the analyzed gliflozins, we correlated the R_MW_ and log k_W_ values of the six reference substances (standards) with their respective log P values from the literature [19]. This analysis facilitated the development of calibration equations, with sufficient linearity that was confirmed by high correlation coefficients (all r values above 0.9), significant *p*-values, low standard errors of estimates (s), and high Fisher F-distribution values (F). Using these calibration equations, the chromatographic systems were evaluated by calculating the log P_EXP_ values for the standards. The differences between the calculated log P_EXP_ values and the corresponding log P values from the literature did not exceed ±0.32 for S1, ±0.10 for S2, ±0.08 for S3, ±0.11 for S4, ±0.16 for S5, and ±0.06 for S6. Thus, the obtained linear relationships indicate that R_MW_ and log k_W_ are reliable alternatives for determining the partition coefficients of the tested drugs (Table 1).

In the subsequent analysis, the log P values for the examined drugs (log P_EXP_) were derived from the established linear equations. The results of these calculations are presented in Table 2. Notably, the lipophilicity values for CANA, DAPA, EMPA, ERTU, and SOTA were consistently higher when methanol was used as the organic modifier compared to acetonitrile, for both TLC and HPLC techniques. When considering all stationary phases, the lipophilicity values obtained from HPLC exhibited less variability compared to those from TLC. Specifically, the results for the RP8 stationary phase were comparable to those for the RP18 phase, while the values obtained from the more polar CN plates were more diverse. This variation highlights the influence of stationary-phase polarity on the assessment of lipophilicity.

For comparative analysis, the log P values for CANA, DAPA, EMPA, ERTU, and SOTA were also calculated using various computational software types, including iLOGP, MLOGP, SILICOS-IT, XLOGP3, WLOGP, Consensus.LogP, and ALOGP. A notable observation is the variability in log P values for DAPA, EMPA, and ERTU across the different computational models. Conversely, the MLOGP algorithm yielded relatively low lipophilicity values for all tested drugs. The results of these computations are summarized in Table 3.

Table 4 presents the correlation matrix for the log P_EXP_ values obtained for the analyzed drugs from HPLC and TLC methods, as well as the log P values from computational methods. Analysis of experimental lipophilicity values from both chromatographic techniques shows that most correlation coefficients (r) ranged from 0.61 to 0.99, with only two exceptions below this range. Among these values, the highest correlation was found between those from HPLC CN/methanol and TLC CN/acetonitrile, with an r value of 0.9706, while the lowest correlation was between values from HPLC RP8/acetonitrile and TLC CN/methanol, with an r value of 0.7398. The strongest correlations were observed across log P_EXP_ from HPLC methods, with all r values at or above 0.95. High correlations were noted within values from RP8/methanol and RP18/methanol, RP8/acetonitrile and RP18/acetonitrile, RP8/methanol and RP8/acetonitrile, and RP8/methanol and RP18/acetonitrile. On the other hand, the weakest correlations were observed between values obtained in HPLC CN/acetonitrile and RP18/acetonitrile (r = 0.9492). In contrast, TLC-based lipophilicity values displayed more variability than HPLC ones, with correlation coefficients between 0.59 and 0.99. Significant correlations among TLC methods were observed for the RP systems, particularly between RP8/methanol and RP18/methanol, and between RP8/acetonitrile and RP18/acetonitrile, while the weakest correlations were observed between TLC CN/methanol and RP8/acetonitrile (r = 0.59).

When comparing the results from computational methods, the analysis showed that log P values predicted by the iLOGP and Consensus.Log P algorithms generally had a weak correlation with experimental data. However, some computational values did approximate the experimental results closely. Interestingly, three theoretical methods, i.e., ALOGP, MLOGP, and XLOGP3, showed strong correlations with experimental TLC systems, particularly in RP mode with methanol. Notable correlations included log P values from TLC RP18/methanol and TLC CN/acetonitrile systems with MLOGP, achieving correlation coefficients of 0.9934 and 0.9904, respectively. Further, the computed log P values exhibited considerable variability across these theoretical models. Of particular interest is the lack of correlations between lipophilicity values predicted by the iLOGP algorithm, a physics-based method relying on solvation free energies in octanol and water, and those from the Consensus.Log P algorithm, which averages predictions from five methods, with other theoretical models, i.e., MLOGP, SILICOS-IT, XLOGP3, WLOGP, and ALOGP (Table 4).

To identify the best-correlated values of lipophilicity for CANA, DAPA, EMPA, ERTU, and SOTA, using all the above methods, respective linear equations were derived and are presented in Table 5, along with parameters of descriptive statistics, including significance levels, standard errors of the estimate, and Fisher F-distribution values. The r values above 0.97 along with low *p*-values (<0.007) suggest that the proposed models are both valid and statistically significant.

**Figure 2:**
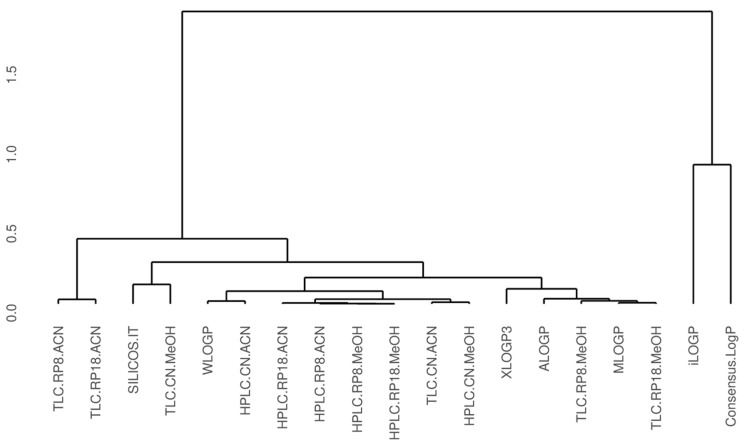
Cluster analysis for chromatographical systems and computational models using lipophilicity for the analyzed drugs (log P_EXP_ and log P) as a similarity measure; ACN—acetonitrile; MeOH—methanol.

In addition, chromatographically determined lipophilicity values for the analyzed drugs were compared with theoretical values using cluster analysis, as shown in the dendrogram in Figure 2. This analysis revealed two main clusters. The first cluster includes all experimental methods (encompassing all TLC and HPLC systems) along with five theoretical methods: SILICOS-IT, WLOGP, XLOGP3, MLOGP, and ALOGP. The second cluster comprises the remaining theoretical methods, iLOGP and Consensus.Log P. Within the first cluster, the TLC RP18/acetonitrile and TLC RP8/acetonitrile systems form a distinct subgroup, setting them apart from the other chromatographic methods. A second subgroup includes the remaining chromatographic methods, except for the TLC RP8 and RP18 systems using methanol. Interestingly, the WLOGP model aligns more closely with the chromatographic methods in this second subgroup than with ALOGP, MLOGP, and XLOGP3, which correlate more with the TLC RP systems using methanol. Among the chromatographic methods in the second subgroup, HPLC reversed-phase systems (RP18 and RP8) cluster together, regardless of the solvent used. Additionally, Figure 2 highlights the distinct separation of iLOGP and Consensus.Log P values from those of other theoretical models within this theoretical cluster.

**Figure 3:**
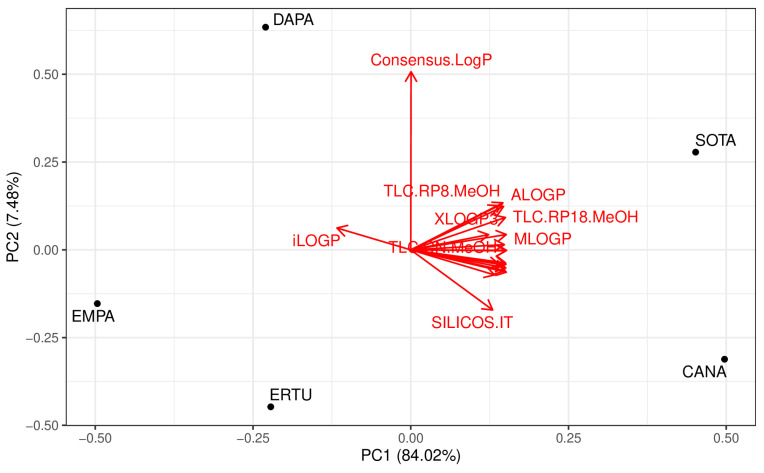
Principal component analysis (PCA) of chromatographical systems and computational models based on lipophilicity similarity of canagliflozin (CANA), dapagliflozin (DAPA), empagliflozin (EMPA), ertugliflozin (ERTU), and sotagliflozin (SOTA); ACN—acetonitrile; MeOH—methanol.

Additionally, the scaled principal component analysis (PCA) was applied to three datasets: log P values from TLC and HPLC experiments, and theoretical log P values for the analyzed drugs. A key finding from this PCA is the high redundancy across both experimental and computational methods, with 84% of the information shared among the datasets. The PCA score plot shows that log P values for CANA, DAPA, EMPA, ERTU, and SOTA from experimental methods closely cluster with each other and with those from computational methods, except for the iLOGP and Consensus.Log P models. Notably, compounds with a higher first principal component (PC1), such as CANA and SOTA, correspond to the highest lipophilicity values, while lower scores align with the lipophilicity of DAPA and ERTU, and the lowest with EMPA. PC1 also reveals that the iLOGP and Consensus.Log P models have the lowest scores, while the other methods cluster closely together. The second principal component (PC2) accounts for an additional 7.5% of the total information. The respective score plot illustrates that all experimental method values form a tightly correlated cluster, also including lipophilicity values from theoretical models—except for Consensus.Log P, which shows a higher score (Figure 3).

Considering the obtained correlations and chemometric evaluations, the standardized chromatographic methods demonstrated greater consistency compared to the computational algorithms used. This result suggests a need for selective method application when determining lipophilicity, tailoring to the specific drug group under analysis.

The hydrophobic effect is a key driving force behind the passive transport of drugs across biological membranes, as well as their binding to receptors. A drug’s lipophilicity significantly influences its protein binding, distribution, and metabolism. Generally, lipophilic compounds tend to be more readily metabolized and often exhibit higher clearance rates. However, high lipophilicity can lead to increased non-specific plasma protein binding, which may result in adverse effects, toxicity, and a poor ADME (absorption, distribution, metabolism, excretion) profile [3].

**Figure 4:**
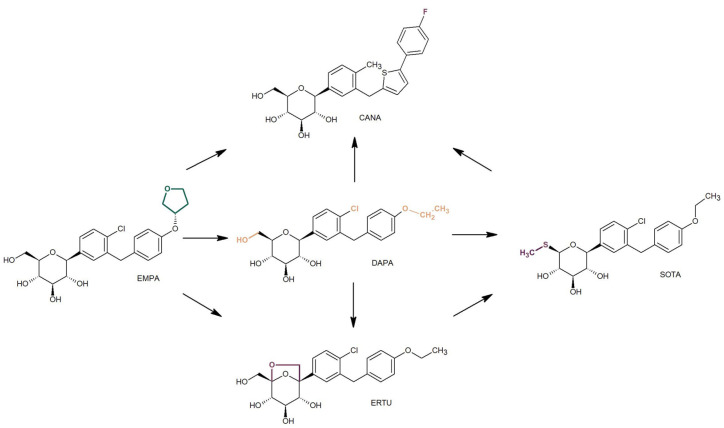
Structural evolution of dapagliflozin (DAPA) and selected gliflozins: canagliflozin (CANA), empagliflozin (EMPA), ertugliflozin (ERTU), and sotagliflozin (SOTA), highlighting structural modifications affecting lipophilicity. The arrows indicate the direction of increasing lipophilic properties across the drugs.

DAPA is the foundational drug from which other gliflozins were synthesized. Its structure features several elements that enhance its inhibitory effect on SGLT proteins, notably two lipophilic constituents: chloro and ethoxy groups, positioned at the 4 and 4′ locations on the aromatic rings (Figure 4). As a result, DAPA demonstrates exceptional inhibitory activity against SGLT2, with a concentration of drug required for 50% inhibition (IC_50_) of 1.12 nM, while maintaining high selectivity over SGLT1 (IC_50_ = 1390 nM). In contrast, CANA has a heterocyclic component and fluorine substitution in its aglycone structure (Figure 4), which increases its lipophilicity, as confirmed in the present study. Consequently, CANA exhibits higher lipophilicity than DAPA; however, this does not directly translate to greater potency against SGLT2, with an IC_50_ of 2.2 nM and IC_50_ for SGLT1 at 910 nM [15]. EMPA displays lower lipophilicity than DAPA, likely due to the replacement of the more lipophilic aliphatic chain in DAPA with a less lipophilic furan ring (Figure 4). Interestingly, the introduction of this tetrahydrofuran-3-oxy moiety did not alter the SGLT2 inhibitory effect but enhanced selectivity (IC_50_ for SGLT2 = 3.1 nM while IC_50_ for SGLT1 = 8300 nM) compared to DAPA [21,22]. The higher log P value for ERTU than for DAPA is also supported by our experimental findings and may be attributed to modifications in the sugar group structure of ERTU, specifically the introduction of a bicyclic structure (Figure 4). This results in ERTU emerging as the most potent and selective SGLT2 inhibitor, with IC_50_ values of 0.87 nM for SGLT2 and 1960 nM for SGLT1 [21,22].

When the first SGLT inhibitors were introduced as antidiabetic agents, selectivity toward renal SGLT2 was a crucial feature for developing new safe drug candidates. However, recent findings suggest that the concurrent inhibition of both SGLT2 in the kidney and SGLT1 in the gastrointestinal tract may enhance glycemic control, particularly in patients with poor glycemic control. This shift in focus has led to a significant push toward the development of dual SGLT1/SGLT2 inhibitors. SOTA stands out as the first dual inhibitor approved for clinical use and serves as an innovative lead compound for future multitarget antidiabetic drugs. It was synthesized by substituting the 6-methylhydroxy group in DAPA’s sugar moiety with an isosteric methylsulfanyl group (Figure 4) and demonstrates potency toward both SGLT subtypes but exhibits lower selectivity than the other gliflozins, with IC_50_ values of 1.8 nM for SGLT2 and 36 nM for SGLT1 [23,24]. This modification of the sugar group at position 6 also correlates with SOTA’s higher log P value compared to DAPA, EMPA, and ERTU, as evidenced in our experiments.

The chromatographically obtained log P values (log P_EXP_) for the analyzed SGLT inhibitors, i.e., CANA, DAPA, EMPA, ERTU, and SOTA, were also related to their ADME parameters taken from the literature (Table 6). The analyzed drugs share several pharmacokinetic characteristics, including a long elimination half-life (T_0.5_) that allows for once-daily administration (the longest being SOTA), similar apparent clearance rates, and volumes of distribution, with the exception of SOTA, which exhibits notably higher values for both parameters. Additionally, these drugs undergo extensive hepatic metabolism, primarily via glucuronidation, and have low renal elimination as parent drugs. However, they differ somewhat in terms of protein-binding capacity, potency toward SGLT proteins, and required doses [21,22,25]. In particular, the higher lipophilicity of CANA and SOTA compared to DAPA, EMPA, and ERTU, confirmed in the present study, may contribute to increased non-specific plasma protein binding, although it seems not to be strictly connected to respective T_0.5_ values. An interesting trend was observed regarding typical therapeutic doses of individual drugs, with the highest doses for CANA and SOTA, which also exhibit the highest lipophilicity. This finding may be somewhat unexpected, but it suggests that other properties of the analyzed drugs, beyond lipophilicity, also play a significant role [26].

In the literature, the concept of lipophilic ligand efficiency (LLE) has been introduced to normalize drug potency relative to lipophilicity. LLE is calculated as the difference between the negative logarithm of IC_50_ (pIC_50_) and log P. This metric assesses how effectively a drug binds to its target while excluding non-specific entropic factors. By utilizing this tool, we can determine whether increased potency is accompanied by heightened lipophilicity. When the drug exhibits simultaneous increases in both potency and lipophilicity, while maintaining a constant LLE, it suggests that the increase in potency may be attributable to enhanced lipophilicity. In contrast, the specific increase in LLE without an increase in lipophilicity indicates that the improvement in potency might not be strictly related to higher lipophilicity [26,27]. Based on our experimental lipophilicity data, the calculated LLE values, and available literature on the potency of the analyzed gliflozins [21,22,25], some interesting results were obtained. For DAPA, EMPA, and ERTU, all of which present similar LLE values, simultaneous increases in both potency and lipophilicity were observed, while maintaining a constant LLE for SGLT2 protein, confirming that lipophilicity plays a crucial role in their activities. However, taking into account the LLE values obtained for all five gliflozins, it could be suggested that their potency does not have to be strictly related to the increase in lipophilicity, as was seen for CANA and SOTA with the highest log P and lower LLE values (Table 6).

## 3. Materials and Methods

### 3.1. Substances and Solvents

The gliflozin drugs analyzed in this study, i.e., canagliflozin (CANA), dapagliflozin (DAPA), empagliflozin (EMPA), ertugliflozin (ERTU), and sotagliflozin (SOTA), were obtained from abrc GmbH (Karlsruhe, Germany. Six reference compounds (standards) with established lipophilicity values [19] from Sigma-Aldrich (Saint Louis, MO, USA) and Galfarm (Kraków, Poland) were used for comparison: 2-aminophenol (S1, log P 0.62), salicylamide (S2, log P 1.28), 4-dimethylaminobenzaldehyde (S3, log P 1.81), eugenol (S4, log P 2.27), 2-naphthol (S5, log P 2.70), and diphenylamine (S6, log P 3.5). Acetonitrile, methanol, and water, all HPLC grade, were used in the preparation of mobile phases and were supplied by J.T. Baker (Center Valley, PA, USA) and Merck (Darmstadt, Germany).

### 3.2. TLC Method

Plates (10 × 10 cm, 0.25 mm) pre-coated with silica gel RP18F_254_, RP8F_254_ and CNF_254_ from Merck were used as the stationary phases. The tested drugs and six reference substances were dissolved in methanol to obtain working solutions at a concentration of 2 mg/mL. Volumes of 5 µL of these working solutions were spotted onto the plates which were then developed at a distance of 9 cm in the horizontal developing chambers (Chromdes, Lublin, Poland). The ranges for the organic modifier concentration in water varied depending on the type of stationary phase and the modifier used. For RP18 and RP8 systems, acetonitrile in the range 35–60% and methanol in the range 60–85% were used. For CN systems, acetonitrile was used in the range of 40–60% while methanol was used in the range of 45–65%. For all chromatographic systems, a 5% step change in the concentration of the organic modifier was applied (Table S1 in Supplementary Files). The developed plates were observed at 254 nm using a UV lamp from Camag (Muttenz, Switzerland). All experiments were performed in triplicate with a constant temperature of 23 ± 0.5 °C.

### 3.3. HPLC Method

All experiments were performed using an HPLC system equipped with a solvent delivery pump 515 and a UV/VIS detector 2487 from Waters UK Sales (Elstree, UK), a degasser 964 from Jasco Inc. (Easton, MD, USA), and LiChrospher RP18, RP8 and CN columns (125 × 4.0 mm, 5 µm) from Merck. For RP18 and RP8 stationary phases, acetonitrile was used in the range of 35–60% and methanol in the range of 55–80%. For CN adsorbent, acetonitrile was used in the range 30–55% and methanol in the range 45–70%. For all chromatographic systems, a 5% step change in the concentration of the organic modifier was applied (Table S2 in Supplementary Files). The flow for each mobile phase and each chromatographic run was 1 mL/min while detection was performed at 260 nm. The analyzed drugs and six reference substances were dissolved in methanol to obtain concentrations of 0.1 mg/mL and injected into the column in the volume of 20 µL. All experiments were performed in triplicate with a constant temperature of 23 ± 0.5 °C.

### 3.4. Standardization Procedure

In our study, six standards of known lipophilicity in the range of 0.62–3.5 were used. For each standard, the same chromatographic procedures were performed as for the analyzed drugs, obtaining their R_F_ or t_R_ values. Then, the R_M_, R_MW_, log k, and log k_W_ values were calculated using Equations (1)–(3). In the next step, correlations between the chromatographically obtained R_MW_ or log k_W_ values for these six standards and their literary log P values [19] were calculated and expressed as respective calibration equations (Table 1). In addition, using these calibration equations, the chromatographic systems were evaluated by calculating the log P_EXP_ values for the standards. The differences between the calculated log P_EXP_ values and the corresponding log P values from the literature did not exceed ±0.32 for S1, ±0.10 for S2, ±0.08 for S3, ±0.11 for S4, ±0.16 for S5, and ±0.06 for S6. From these calibration equations, the experimental log P_TLC_ and log P_HPLC_ (log P_EXP_) for the examined drugs were determined, using their R_MW_ and log k_W_ values.

### 3.5. Computational Methods

First, five models from SwissADME [17], namely XLOGP3, WLOGP, MLOGP, SILICOS-IT, and iLOGP, were used. XLOGP3 is an atomistic approach with respective corrective factors and a knowledge-based library [28,29]. The next model WLOGP [28] is also an atomistic method, but it is purely applied to a fragmental system. MLOGP, i.e., Moriguchi octanol–water partition coefficient, is calculated from the Moriguchi log P model consisting of a regression equation based on 13 structural parameters, using a training set of 1230 organic molecules, including general aliphatic, aromatic, and heterocyclic compounds [29,30,31]. SILICOS-IT is a hybrid method that relies on 27 fragments and seven topological descriptors [29], while iLOGP is a physics-based method that uses the generalized Born (GB) and solvent-accessible surface area (SA) models to calculate solvation free energies in octanol and water [29,31]. Finally, Consensus.logP is calculated as the arithmetic mean of the values predicted by the five above methods [17,29]. In addition, the ALOGP algorithm consisting of a regression equation based on the hydrophobicity contribution of 115 atom types [32] was used from VCCLAB.org [18].

### 3.6. Statistic and Chemometric Analyses

Statistica 13 from Tibco Software (Palo Alto, CA, USA) was used for the regression and correlation analyses. The principal component analysis (PCA) and the cluster analysis were performed using R (version 4.3.3) with the built-in functions for the computation and “ggplot2” package for the generation of respective plots.

## 4. Conclusions

This study successfully determined the lipophilicity of five oral antidiabetic drugs from the gliflozin class, i.e., canagliflozin (CANA), dapagliflozin (DAPA), empagliflozin (EMPA), ertugliflozin (ERTU), and sotagliflozin (SOTA), using both TLC and HPLC methods. We employed their R_MW_ and log k_W_ parameters for the experimental assessment, supplemented by several computational methods. To evaluate the chromatographic experiments, we implemented a standardization procedure utilizing six reference substances with established lipophilicity values. In addition, chromatographically determined lipophilicity values were compared with theoretical values using cluster analysis as well as principal component analysis (PCA). Our findings indicate that the experimental lipophilicity values exhibit strong correlations, supporting the reliability of R_MW_ and log k_W_ as effective parameters for assessing lipophilicity for these important drugs. In contrast, the lipophilicity values derived from the computational methods displayed lower correlations, both among themselves and when compared to experimental data. Lower correlation values observed for computational methods may be attributed to their inherent limitations, particularly when assessing lipophilicity in complex molecules. This research addresses a gap in the literature, as no previous studies have used chromatographic methods to determine the lipophilicity of gliflozins. What is more, the obtained experimental lipophilicity values were also related to some pharmacological parameters for the analyzed drugs. We believe these findings are significant and establish a valuable foundation for designing future gliflozin derivatives with improved therapeutic profiles. Specifically, our results allow for a confident proposal of a model to experimentally determine the lipophilicity of gliflozin drugs, including new derivatives, using HPLC in the RP18 system with methanol as the mobile phase.

## Figures and Tables

**Table 1:** Calibration equations based on R_MW_ or log k_W_ and the literary lipophilicity of the standards.

Stationary Phase	Methanol	Acetonitrile
TLC.RP18	log P_EXP_ = 1.118 × R_MW_ − 0.784 F = 355.286, r = 0.9944, *p* = 0.0000, s = 0.1077	log P_EXP_ = 1.647 × R_MW_ − 1.438 F = 121.518, r = 0.9839, *p* = 0.0004, s = 0.1223
TLC.RP8	log P_EXP_ = 1.501 × R_MW_ − 1.244 F = 120.465, r = 0.9838, *p* = 0.0004, s = 0.1349	log P_EXP_ = 1.950 × R_MW_ − 1.626 F = 83.902; r = 0.9769, *p* = 0.0008, s = 0.1226
TLC.CN	log P_EXP_ = 2.619 × R_MW_ − 1.511 F = 72.472, r = 0.9735, *p* = 0.001, s = 0.0975	log P_EXP_ = 1.138 × R_MW_ − 0.506 F = 40.627, r = 0.9104, *p* = 0.0031, s = 0.2881
HPLC.RP18	log P_EXP_ = 1.071 × log k_W_ + 0.264 F = 48.296, r = 0.9235, *p* = 0.0023, s = 0.2847	log P_EXP_ = 1.601 × log k_W_ − 0.449 F = 42.389, r = 0.9559, *p* = 0.0029, s = 0.2011
HPLC.RP8	log P_EXP_ = 1.133 × log k_W_ + 0.161 F = 50.679, r = 0.9627, *p* = 0.0021, s = 0.2638	log P_EXP_ = 1.360 × log k_W_ − 0.069 F = 47.725, r = 0.9606, *p* = 0.0023, s = 0.2253
HPLC.CN	log P_EXP_ = 2.642 × log k_W_ − 1.001 F = 40.704, r = 0.9542, *p* = 0.0031, s = 0.1239	log P_EXP_ = 2.521 × log k_W_ − 0.932 F = 41.742, r = 0.9553, *p* = 0.003, s = 0.1285

R_MW_—R_M_ value extrapolated to 100% water in the mobile phase; log k_W_—retention factor for a mobile phase containing 100% water; r—correlation coefficient; *p*—significance value; s—standard error of estimate; F—Fisher distribution value.

**Table 2:** The log P_EXP_ of the analyzed drugs obtained using TLC and HPLC methods with a standardization procedure.

Methods	CANA	DAPA	EMPA	ERTU	SOTA
HPLC.CN/ACN	3.73	2.34	2.30	2.49	3.59
HPLC.CN/MeOH	4.91	3.08	2.66	3.36	4.57
HPLC.RP18/ACN	3.28	2.00	1.35	2.22	3.40
HPLC.RP18/MeOH	4.55	3.27	2.85	3.59	4.50
HPLC.RP8/ACN	3.23	2.11	1.70	2.40	3.28
HPLC.RP8/MeOH	4.43	3.20	2.78	3.49	4.43
TLC.CN/ACN	2.40	2.03	1.87	2.01	2.32
TLC.CN/MeOH	5.86	4.72	3.51	3.97	4.61
TLC.RP18/ACN	3.47	2.22	1.30	3.13	3.69
TLC.RP18/MeOH	4.43	3.58	2.99	3.27	4.50
TLC.RP8/ACN	3.55	2.30	1.68	2.99	4.08
TLC.RP8/MeOH	4.07	3.14	2.48	3.54	4.26

log P_EXP_—log P values derived for the analyzed drugs from the established linear equations and respective R_MW_ or log k_W_ values; ACN—acetonitrile; MeOH—methanol; CANA—canagliflozin; DAPA—dapagliflozin; EMPA—empagliflozin; ERTU—ertugliflozin; SOTA—sotagliflozin.

**Table 3:** The log P values of the analyzed drugs using computational methods.

Methods	CANA	DAPA	EMPA	ERTU	SOTA
iLOGP	3.27	3.12	3.18	3.02	2.88
XLOGP	3.23	2.35	2.03	1.71	3.23
WLOGP	3.06	1.52	1.29	1.25	2.85
MLOGP	1.95	1.07	0.70	0.89	1.88
SILICOS-IT	4.83	2.77	2.66	3.00	3.48
Consensus.LogP	3.26	2.18	1.97	1.97	2.86
ALOGP	3.09	2.52	1.79	2.22	3.19

CANA—canagliflozin; DAPA—dapagliflozin; EMPA—empagliflozin; ERTU—ertugliflozin; SOTA—sotagliflozin.

**Table 4:** Correlation matrix (r) of log P_EXP_ obtained for the analyzed drugs using TLC, HPLC, and log P obtained using computational methods.

	ALOGP	Consensus LogP	iLOGP	MLOGP	SILICOS-IT	WLOGP	XLOGP3	HPLC CN ACN	HPLC CN MeOH	HPLC RP18 ACN	HPLC RP18 MeOH	HPLC RP8 ACN	HPLC RP8 MeOH	TLC CN ACN	TLC CN MeOH	TLC RP18 ACN	TLC RP18 MeOH	TLC RP8 ACN	TLC RP8 MeOH
ALOGP	1																		
Consensus.LogP	0.2529	1																	
iLOGP	–0.6506	0.1233	1																
MLOGP	0.9684	0.0750	0.7576	1															
SILICOS-IT	0.7373	–0.3705	0.5396	0.8423	1														
WLOGP	0.9185	0.0023	0.8029	0.9872	0.8624	1													
XLOGP3	0.9056	0.2062	0.7582	0.9560	0.7560	0.9708	1												
HPLC.CN/ACN	0.8980	–0.1262	0.8422	0.9738	0.8763	0.9830	0.9141	1											
HPLC.CN/MeOH	0.9275	–0.1214	0.7528	0.9736	0.8970	0.9587	0.8736	0.9835	1										
HPLC.RP18/ACN	0.9621	0.0197	0.7466	0.9627	0.7913	0.9217	0.8498	0.9492	0.9775	1									
HPLC.RP18/MeOH	0.9373	–0.0799	0.7541	0.9585	0.8378	0.9269	0.8371	0.9638	0.9904	0.9947	1								
HPLC.RP8/ACN	0.9422	–0.0477	0.7633	0.9555	0.8095	0.9201	0.8347	0.9574	0.9834	0.9976	0.9987	1							
HPLC.RP8/MeOH	0.9424	–0.0591	0.7631	0.9613	0.8265	0.9293	0.8441	0.9643	0.9886	0.9966	0.9996	0.9995	1						
TLC.CN/ACN	0.9617	0.0063	0.7076	0.9904	0.8856	0.9692	0.9115	0.9706	0.9904	0.9739	0.9772	0.9709	0.9768	1					
TLC.CN/MeOH	0.8223	0.0501	0.2932	0.8298	0.8778	0.8024	0.7772	0.7518	0.8107	0.7539	0.7614	0.7398	0.7544	0.8624	1				
TLC.RP18/ACN	0.8488	0.0735	0.5921	0.8060	0.6725	0.7289	0.6067	0.8042	0.8786	0.9339	0.9310	0.9378	0.9295	0.8527	0.6233	1			
TLC.RP18/MeOH	0.9879	0.1778	0.7359	0.9934	0.7835	0.9672	0.9519	0.9470	0.9525	0.9640	0.9483	0.9502	0.9533	0.9784	0.8140	0.8149	1		
TLC.RP8/ACN	0.8885	0.0276	0.7560	0.8726	0.6723	0.8183	0.7204	0.8818	0.9167	0.9692	0.9623	0.9726	0.9651	0.8906	0.5903	0.9724	0.8805	1	
TLC.RP8/MeOH	0.9702	0.2483	0.7515	0.9815	0.7393	0.9662	0.9787	0.9276	0.9143	0.9245	0.9033	0.9066	0.9107	0.9492	0.7849	0.7375	0.9911	0.8271	1

r—correlation coefficient; ACN—acetonitrile; MeOH—methanol.

**Table 5:** Descriptive statistics for the best correlated systems for assessing lipophilicity of the analyzed drugs.

Correlations	r	F	*p*	s
MLOGP—ALOGP	0.9684	45.16	0.0067	0.1668
WLOGP—MLOGP	0.9872	114.9	0.0017	0.1632
XLOGP3—WLOGP	0.9708	49.19	0.0060	0.1924
ALOGP—TLC.RP18/MEOH	0.9879	122.2	0.0016	0.1219
ALOGP—TLC.RP8/MEOH	0.9702	48.05	0.0062	0.2338
MLOGP—TLC.CN/ACN	0.9904	153.6	0.0011	0.0358
MLOGP—TLC.RP18/MEOH	0.9934	224.4	0.0006	0.0905
XLOGP3—TLC.RP8/MEOH	0.9787	68.25	0.0037	0.1978
WLOGP—HPLC.CN/ACN	0.9830	85.88	0.0027	0.1502
HPLC.CN/MEOH—HPLC.CN/ACN	0.9835	88.68	0.0025	0.2036
HPLC.RP18/CAN—HPLC.RP8/ACN	0.9976	620.7	0.0001	0.0700
HPLC.RP8/CAN—HPLC.RP18/MeOH	0.9987	1178	0.0000	0.0405
HPLC.RP8/MeOH—HPLC.RP18/MeOH	0.9996	418	0.0000	0.0229
HPLC.RP8/MeOH—HPLC.RP8/ACN	0.9995	2999	0.0000	0.0271
HPLC.CN/MeOH—TLC.CN/ACN	0.9904	153.5	0.0011	0.1558
HPLC.RP8/MeOH—TLC.CN/ACN	0.9768	62.49	0.0042	0.0554
TLC.RP18/MeOH—TLC.CN/ACN	0.9784	67.14	0.0038	0.1629
TLC.RP8/CAN—TLC.RP18/ACN	0.9724	52.12	0.0055	0.2670
TLC.RP8/MeOH—TLC.RP18/MeOH	0.9911	166.4	0.0010	0.1283

r—correlation coefficient; *p*—significance value; s—standard error of estimate; F—Fisher distribution value; ACN—acetonitrile; MeOH—methanol.

**Table 6:** Experimental lipophilicity from HPLC and TLC methods (log P_EXP_), and pharmacological properties of the analyzed drugs [21,22,25].

Drug	log P_EXP_ (Mean ± SD)	IC_50_ SGLT2/SGLT1 (nM)	PB (%)	CL (L/h)	VD (L)	LLE	T_0.5_ (h)	Dose (mg)
CANA	3.99 ± 0.91	2.2/910	99	11.52	83.5	4.67	10.6	100–300
SOTA	3.94 ± 0.67	1.8/36	98	261	9392	4.8	21	200–400
ERTU	3.04 ± 0.61	0.87/1960	94	10.68	85.5	6.02	11	5–15
DAPA	2.83 ± 0.78	1.12/1390	91	12.42	118	6.12	12.9	5–50
EMPA	2.28 ± 0.73	3.1/8300	86	10.6	73.8	6.23	12.4	10–25

SD—standard deviation; IC_50_—the concentration of drug required for 50% inhibition; SGLT2/SGLT1—sodium–glucose transporters 2/1; PB—protein-binding capacity; CL—apparent total clearance; VD—apparent volume of distribution; LLE—lipophilic ligand efficiency; T_0.5_—the half-life.

## Data Availability

Data are contained within the article and Supplementary Materials.

## Supplementary Materials

The following supporting information can be downloaded at: https://www.mdpi.com/article/10.3390/molecules30010115/s1, Table S1. The R_F_ values of the analyzed drugs and standards of known lipophilicity obtained using the TLC method; Table S2. The log k values of the analyzed drugs and standards of known lipophilicity obtained using the HPLC method; Table S3. The R_MW_ values of the analyzed drugs obtained using the TLC method; Table S4. The R_MW_ values of the standards of known lipophilicity obtained using the TLC method; Table S5. The log k_W_ values of the analyzed drugs obtained using the HPLC method; Table S6. The log k_W_ values of the standards of known lipophilicity obtained using the HPLC method.
