# Supplementary material for: Standardized Chromatographic and Computational Approaches for Lipophilicity Analysis of Five Gliflozin Antidiabetic Drugs in Relation to Their Biological Activity
Source: Molecules. 2024 Dec 31;30(1):115. doi: 10.3390/molecules30010115 (PMC11721022; doi:10.3390/molecules30010115)
Supplement: Supplementary file 1 [file molecules-30-00115-s001.zip › Supplementary Tables S1-S2.pdf]

Table S1. The R_F values of the analyzed drugs and standards of known lipophilicity obtained using TLC method.

%	ACETONITRILE											METHANOL										
R_F values in the RP18 systems																						
	**CANA**	**DAPA**	**EMPA**	**ERTU**	**SOTA**	**S1**	**S2**	**S3**	**S4**	**S5**	**S6**	**CANA**	**DAPA**	**EMPA**	**ERTU**	**SOTA**	**S1**	**S2**	**S3**	**S4**	**S5**	**S6**
**35**	0.06	0.17	0.27	0.10	0.04	0.20	0.28	0.11	0.08	0.07	0.03											
**40**	0.17	0.32	0.43	0.29	0.11	0.25	0.42	0.18	0.14	0.18	0.06											
**45**	0.33	0.53	0.61	0.48	0.24	0.29	0.54	0.24	0.24	0.25	0.11											
**50**	0.47	0.62	0.66	0.59	0.33	0.34	0.59	0.31	0.30	0.36	0.15											
**55**	0.55	0.67	0.71	0.71	0.42	0.39	0.67	0.37	0.37	0.47	0.20											
**60**	0.64	0.78	0.79	0.78	0.51	0.44	0.77	0.46	0.44	0.58	0.25	0.06	0.12	0.18	0.11	0.04	0.29	0.50	0.24	0.21	0.20	0.08
**65**												0.10	0.22	0.30	0.19	0.09	0.37	0.57	0.33	0.30	0.29	0.13
**70**												0.16	0.27	0.38	0.27	0.13	0.41	0.61	0.38	0.33	0.39	0.17
**75**												0.29	0.42	0.49	0.40	0.24	0.44	0.69	0.47	0.46	0.48	0.28
**80**												0.44	0.56	0.64	0.53	0.33	0.48	0.78	0.56	0.56	0.63	0.39
**85**												0.63	0.74	0.77	0.77	0.56	0.53	0.83	0.68	0.71	0.72	0.56
R_F values in the RP8 systems																						
**35**	0.10	0.22	0.26	0.16	0.06	0.20	0.36	0.16	0.13	0.13	0.05											
**40**	0.22	0.39	0.45	0.31	0.17	0.25	0.46	0.21	0.19	0.20	0.08											
**45**	0.42	0.52	0.60	0.50	0.27	0.30	0.54	0.29	0.24	0.28	0.13											
**50**	0.51	0.63	0.67	0.61	0.41	0.35	0.64	0.38	0.38	0.42	0.19											
**55**	0.64	0.72	0.73	0.71	0.56	0.40	0.69	0.44	0.47	0.50	0.26											
**60**	0.70	0.79	0.79	0.78	0.59	0.45	0.78	0.50	0.53	0.58	0.32	0.13	0.22	0.30	0.20	0.11	0.46	0.48	0.26	0.29	0.30	0.14
**65**												0.27	0.41	0.48	0.39	0.23	0.51	0.63	0.42	0.45	0.45	0.29
**70**												0.39	0.50	0.55	0.51	0.37	0.59	0.70	0.47	0.51	0.55	0.38
**75**												0.45	0.56	0.61	0.53	0.39	0.65	0.74	0.53	0.56	0.62	0.46
**80**												0.61	0.70	0.73	0.69	0.58	0.69	0.79	0.64	0.69	0.70	0.56
**85**												0.72	0.78	0.80	0.78	0.69	0.73	0.83	0.69	0.77	0.79	0.69
R_F values in the CN systems																						
**40**	0.21	0.27	0.29	0.27	0.22	0.41	0.45	0.29	0.24	0.24	0.10											
**45**	0.48	0.51	0.51	0.53	0.44	0.54	0.58	0.41	0.35	0.38	0.17	0.17	0.29	0.31	0.27	0.22	0.49	0.54	0.42	0.44	0.37	0.26
**50**	0.64	0.67	0.68	0.67	0.58	0.60	0.65	0.52	0.44	0.50	0.31	0.32	0.42	0.47	0.44	0.33	0.56	0.60	0.51	0.54	0.46	0.35
**55**	0.71	0.72	0.72	0.73	0.69	0.67	0.74	0.68	0.66	0.64	0.53	0.42	0.53	0.56	0.52	0.43	0.60	0.68	0.58	0.60	0.54	0.42
**60**	0.78	0.79	0.78	0.79	0.76	0.72	0.79	0.77	0.77	0.74	0.66	0.56	0.61	0.66	0.61	0.56	0.65	0.73	0.62	0.67	0.63	0.50
**65**												0.67	0.78	0.71	0.72	0.64	0.71	0.77	0.70	0.79	0.72	0.60

2-Aminophenol (S1), Salicylamide (S2), 4-dimethylaminobenzaldehyde (S3), Eugenol (S4), 2-Naphtol (S5), Diphenylamine (S6)

Table S2. The log k values of the analyzed drugs and standards of known lipophilicity obtained using HPLC method.

%	ACETONITRILE											METHANOL										
	log k values in the RP18 systems																					
	**CANA**	**DAPA**	**EMPA**	**ERTU**	**SOTA**	**S1**	**S2**	**S3**	**S4**	**S5**	**S6**	**CANA**	**DAPA**	**EMPA**	**ERTU**	**SOTA**	**S1**	**S2**	**S3**	**S4**	**S5**	**S6**
**35**	1.15	0.72	0.53	0.83	1.20	0.57	0.41	0.71	0.87	0.84	1.36											
**40**	0.80	0.46	0.39	0.56	0.88	0.50	0.31	0.57	0.69	0.7	1.26											
**45**	0.56	0.30	0.30	0.39	0.65	0.47	0.23	0.47	0.55	0.53	1.03											
**50**	0.40	0.19	0.19	0.27	0.49	0.42	0.17	0.36	0.41	0.43	0.84											
**55**	0.28	0.11	0.11	0.20	0.36	0.35	0.11	0.31	0.31	0.27	0.68	1.39	0.90	0.76	1.06	1.41	0.10	0.14	0.55	0.62	0.62	1.15
**60**	0.19	0.05	0.09	0.13	0.25	0.28	-0.03	0.25	0.23	0.21	0.53	1.09	0.75	0.54	0.81	1.13	0.02	0.03	0.42	0.46	0.49	0.94
**65**												0.80	0.60	0.36	0.58	0.85	0.01	-0.01	0.30	0.31	0.33	0.74
**70**												0.55	0.34	0.19	0.37	0.61	-0.09	-0.06	0.20	0.18	0.21	0.56
**75**												0.37	0.21	0.08	0.23	0.43	-0.10	-0.08	0.13	0.08	0.13	0.42
**80**												0.18	0.07	-0.01	0.10	0.23	-0.14	-0.16	0.06	-0.02	0.05	0.26
	log k values in the RP8 systems																					
**35**	1.17	0.78	0.59	0.88	1.24	0.44	0.38	0.78	0.93	0.93	1.49											
**40**	0.80	0.53	0.47	0.59	0.90	0.34	0.29	0.63	0.74	0.74	1.31											
**45**	0.56	0.42	0.28	0.41	0.67	0.30	0.25	0.52	0.60	0.60	1.09											
**50**	0.38	0.23	0.15	0.28	0.49	0.24	0.16	0.42	0.47	0.47	0.90											
**55**	0.27	0.19	0.11	0.19	0.37	0.20	0.12	0.35	0.37	0.38	0.75	1.31	1.02	0.78	1.03	1.35	0.15	0.24	0.68	0.70	0.67	1.11
**60**	0.14	0.13	0.08	0.11	0.26	0.17	0.09	0.28	0.27	0.29	0.63	0.99	0.72	0.55	0.76	1.04	0.13	0.14	0.54	0.53	0.52	0.89
**65**												0.72	0.58	0.37	0.55	0.78	0.06	0.09	0.43	0.39	0.39	0.69
**70**												0.50	0.42	0.23	0.38	0.56	0.02	0.04	0.34	0.27	0.28	0.53
**75**												0.30	0.29	0.12	0.24	0.36	-0.01	0	0.26	0.16	0.19	0.37
**80**												0.16	0.20	0.05	0.12	0.21	-0.04	-0.03	0.20	0.08	0.12	0.25
	log k values in the CN systems																					
**30**	0.98	0.71	0.59	0.74	0.97	0.53	0.43	0.60	0.69	0.75	1.13											
**35**	0.72	0.51	0.42	0.54	0.74	0.43	0.38	0.50	0.58	0.62	0.94											
**40**	0.50	0.42	0.28	0.38	0.54	0.34	0.30	0.42	0.47	0.51	0.82											
**45**	0.36	0.27	0.17	0.27	0.41	0.32	0.23	0.36	0.39	0.42	0.70	0.88	0.62	0.55	0.65	0.85	0.42	0.38	0.46	0.43	0.59	0.80
**50**	0.25	0.20	0.15	0.20	0.30	0.29	0.15	0.29	0.31	0.33	0.57	0.76	0.54	0.48	0.56	0.74	0.37	0.28	0.41	0.37	0.50	0.72
**55**	0.18	0.17	-0.07	0.17	0.22	0.25	0.07	0.24	0.24	0.25	0.48	0.49	0.35	0.31	0.37	0.50	0.35	0.19	0.31	0.25	0.37	0.57
**60**												0.34	0.26	0.22	0.25	0.36	0.31	0.15	0.25	0.18	0.28	0.48
**65**												0.23	0.17	0.15	0.17	0.25	0.26	0.12	0.20	0.12	0.21	0.36
**70**												0.16	0.13	0.11	0.12	0.18	0.20	0.09	0.17	0.08	0.16	0.27

2-Aminophenol (S1), Salicylamide (S2), 4-dimethylaminobenzaldehyde (S3), Eugenol (S4), 2-Naphtol (S5), Diphenylamine (S6)
