# Supplementary material for: Standardized Chromatographic and Computational Approaches for Lipophilicity Analysis of Five Gliflozin Antidiabetic Drugs in Relation to Their Biological Activity
Source: Molecules. 2024 Dec 31;30(1):115. doi: 10.3390/molecules30010115 (PMC11721022; doi:10.3390/molecules30010115)
Supplement: Supplementary file 1 [file molecules-30-00115-s001.zip › Supplementary Tables S3-S6.pdf]

Table S3. The R_{MW} values of the analyzed drugs obtained using TLC method.

	RP18				RP8				CN			
	R_{MW}	– S	φ	r^2	R_{MW}	– S	φ	r^2	R_{MW}	– S	φ	r^2
CANA												
Acetonitrile	2.98	0.06	53.17	0.9386	2.66	0.05	50.71	0.9521	2.56	0.05	47.84	0.9151
Methanol	4.67	0.06	81.63	0.9955	3.54	0.05	75.84	0.9778	2.82	0.05	58.07	0.9864
DAPA												
Acetonitrile	2.22	0.05	47.13	0.9509	1.99	0.04	45.56	0.9787	2.23	0.05	46.35	0.9253
Methanol	3.91	0.05	77.21	0.9869	2.92	0.04	71.22	0.9700	2.38	0.04	53.79	0.9797
EMPA												
Acetonitrile	1.66	0.04	43.60	0.9443	1.70	0.04	43.45	0.9446	2.09	0.05	45.97	0.9178
Methanol	3.38	0.05	74.26	0.9920	2.48	0.04	68.31	0.9750	1.92	0.04	52.89	0.9683
ERTU												
Acetonitrile	2.77	0.06	48.32	0.9434	2.36	0.05	47.28	0.9687	2.21	0.05	46.09	0.9149
Methanol	3.62	0.05	77.18	0.9315	2.52	0.04	70.61	0.9754	2.09	0.04	54.20	0.9517
SOTA												
Acetonitrile	3.11	0.05	57.40	0.9398	2.93	0.05	54.35	0.9493	2.49	0.05	48.69	0.9558
Methanol	4.73	0.06	84.10	0.9890	3.67	0.05	77.31	0.9685	2.34	0.04	58.11	0.9940

R_{MW}-R_M value extrapolated to 100% water in the mobile phase; S and φ-parameters from the equation $R_M = R_{MW} – S \times \varphi$.

Table S4. The R_{MW} values of the standards of known lipophilicity obtained using TLC method.

	RP18				RP8				CN			
	R_{MW}	– S	φ	r^2	R_{MW}	– S	φ	r^2	R_{MW}	– S	φ	r^2
2-Aminophenol (S1)												
Acetonitrile	1.28	0.02	64.95	0.9973	1.30	0.02	63.64	0.9959	1.21	0.03	44.14	0.9740
Methanol	1.32	0.02	81.36	0.9677	1.30	0.02	63.17	0.9910	0.89	0.02	45.57	0.9928
Salicylamide (S2)												
Acetonitrile	1.57	0.03	45.07	0.9785	1.32	0.03	42.68	0.9948	1.37	0.03	41.75	0.9892
Methanol	1.70	0.03	61.13	0.9809	1.58	0.03	58.44	0.9614	0.98	0.02	41.88	0.9931
4-dimethylaminobenzaldehyde (S3)												
Acetonitrile	1.98	0.03	61.57	0.9859	1.74	0.03	58.75	0.9882	2.24	0.05	48.53	0.9963
Methanol	2.40	0.03	75.88	0.9891	2.19	0.03	72.48	0.9617	1.21	0.02	50.06	0.9871
Eugenol (S4)												
Acetonitrile	2.30	0.04	61.03	0.9631	2.10	0.04	57.37	0.9853	2.62	0.05	50.24	0.9834
Methanol	2.82	0.04	76.22	0.9760	2.38	0.03	69.96	0.9730	1.55	0.03	48.50	0.9719
2-Naphtol (S5)												
Acetonitrile	2.67	0.05	55.94	0.9691	2.18	0.04	55.27	0.9890	2.37	0.05	50.04	0.9967
Methanol	3.03	0.04	74.88	0.9968	2.44	0.04	68.80	0.9852	1.66	0.03	52.41	0.9983
Diphenylamine (S6)												
Acetonitrile	2.83	0.04	69.88	0.9623	2.60	0.04	67.21	0.9906	3.56	0.06	55.12	0.9940
Methanol	3.83	0.05	83.71	0.9912	3.19	0.04	76.52	0.9701	1.82	0.03	59.49	0.9962

R_{MW}-R_M value extrapolated to 100% water in the mobile phase; S and φ-parameters from the equation $R_M = R_{MW} – S \times \varphi$.

Table S5. The log kw values of the analyzed drugs obtained using HPLC method.

	RP18				RP8				CN			
	log kw	– S	φ	r^2	log kw	– S	φ	r^2	log kw	– S	φ	r^2
CANA												
Acetonitrile	2.33	0.04	62.63	0.9397	2.43	0.04	61.50	0.9493	1.85	0.03	58.14	0.9505
Methanol	4.00	0.05	82.54	0.9885	3.77	0.05	81.96	0.9821	2.24	0.03	73.05	0.9663
DAPA												
Acetonitrile	1.53	0.03	59.34	0.9293	1.60	0.03	62.24	0.9310	1.30	0.02	60.09	0.9474
Methanol	2.80	0.03	81.44	0.9908	2.69	0.03	84.38	0.9637	1.54	0.02	74.03	0.9649
EMPA												
Acetonitrile	1.12	0.02	62.50	0.9587	1.30	0.02	60.40	0.9303	1.28	0.02	53.10	0.9661
Methanol	2.41	0.03	77.81	0.9768	2.31	0.03	79.55	0.9641	1.38	0.02	73.63	0.9633
ERTU												
Acetonitrile	1.67	0.03	62.31	0.9245	1.82	0.03	61.25	0.9413	1.36	0.03	59.25	0.9291
Methanol	3.11	0.04	81.20	0.9832	2.93	0.04	81.82	0.9776	1.65	0.02	73.29	0.9651
SOTA												
Acetonitrile	2.40	0.04	64.69	0.9553	2.46	0.04	64.68	0.9542	1.79	0.03	60.37	0.9617
Methanol	3.95	0.05	83.98	0.9905	3.77	0.05	83.33	0.9851	2.11	0.03	74.43	0.9703

log kw-retention factor for a mobile phase containing of 100% water; S and φ-parameters from the equation log k = log kw – S × φ.

Table S6. The log kw values of the standards of known lipophilicity obtained using HPLC method.

	**RP18**				**RP8**				**CN**			
	log kw	– S	φ	r^2	log kw	– S	φ	r^2	log kw	– S	φ	r^2
2-Aminophenol (S1)												
Acetonitrile	0.96	0.01	86.32	0.9844	0.79	0.01	73.79	0.9652	0.80	0.01	77.20	0.9113
Methanol	0.60	0.01	63.81	0.9545	1.30	0.02	63.17	0.9910	0.79	0.01	95.98	0.9876
Salicylamide (S2)												
Acetonitrile	0.99	0.02	59.63	0.9821	0.77	0.01	65.87	0.9719	0.87	0.01	60.63	0.9945
Methanol	0.71	0.01	65.49	0.9527	1.58	0.03	58.44	0.9614	0.85	0.01	75.36	0.9192
4-dimethylaminobenzaldehyde (S3)												
Acetonitrile	1.31	0.02	71.96	0.9704	1.43	0.02	72.86	0.9786	1.00	0.01	71.19	0.9885
Methanol	1.61	0.02	81.46	0.9823	2.19	0.03	72.48	0.9617	1.01	0.01	81.86	0.9664
Eugenol (S4)												
Acetonitrile	1.72	0.03	67.60	0.9831	1.80	0.03	69.25	0.9827	1.21	0.02	67.54	0.9935
Methanol	2.01	0.03	78.02	0.9889	2.38	0.03	69.96	0.9730	1.08	0.01	73.77	0.9751
2-Naphtol (S5)												
Acetonitrile	1.73	0.03	66.70	0.9854	1.76	0.03	70.14	0.9795	1.31	0.02	66.95	0.9903
Methanol	1.86	0.02	80.71	0.9840	2.44	0.04	68.80	0.9852	1.37	0.02	77.22	0.9784
Diphenylamine (S6)												
Acetonitrile	2.59	0.03	74.92	0.9991	2.70	0.04	76.76	0.9902	1.86	0.03	72.60	0.9901
Methanol	3.09	0.04	86.48	0.9939	3.19	0.04	76.52	0.9701	1.78	0.02	81.99	0.9949

log kw-retention factor for a mobile phase containing of 100% water; S and φ-parameters from the equation log k = log kw – S × φ.
